# Identification of 20 polymer types by means of laser-induced breakdown spectroscopy (LIBS) and chemometrics

**DOI:** 10.1007/s00216-021-03622-y

**Published:** 2021-08-30

**Authors:** Zuzana Gajarska, Lukas Brunnbauer, Hans Lohninger, Andreas Limbeck

**Affiliations:** TU Wien, Institute of Chemical Technologies and Analytics, Getreidemarkt 9/164-IAC, 1060 Vienna, Austria

**Keywords:** Polymers, Laser-induced breakdown spectroscopy, Identification, Chemometrics

## Abstract

Over the past few years, laser-induced breakdown spectroscopy (LIBS) has earned a lot of attention in the field of online polymer identification. Unlike the well-established near-infrared spectroscopy (NIR), LIBS analysis is not limited by the sample thickness or color and therefore seems to be a promising candidate for this task. Nevertheless, the similar elemental composition of most polymers results in high similarity of their LIBS spectra, which makes their discrimination challenging. To address this problem, we developed a novel chemometric strategy based on a systematic optimization of two factors influencing the discrimination ability: the set of experimental conditions (laser energy, gate delay, and atmosphere) employed for the LIBS analysis and the set of spectral variables used as a basis for the polymer discrimination. In the process, a novel concept of spectral descriptors was used to extract chemically relevant information from the polymer spectra, cluster purity based on the k-nearest neighbors (k-NN) was established as a suitable tool for monitoring the extent of cluster overlaps and an in-house designed random forest (RDF) experiment combined with a cluster purity–governed forward selection algorithm was employed to identify spectral variables with the greatest relevance for polymer identification. Using this approach, it was possible to discriminate among 20 virgin polymer types, which is the highest number reported in the literature so far. Additionally, using the optimized experimental conditions and data evaluation, robust discrimination performance could be achieved even with polymer samples containing carbon black or other common additives, which hints at an applicability of the developed approach to real-life samples.

**Graphical abstract:**

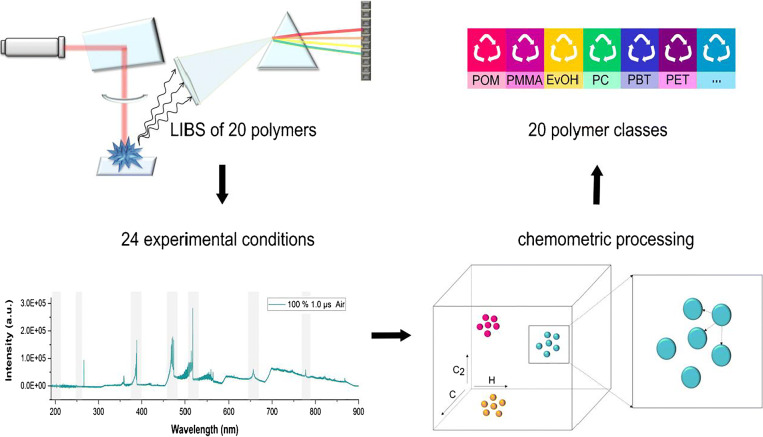

## Introduction

Since the boom of their production in the 1950s, polymers have significantly increased the quality of human life by greatly expanding the availability of everyday products on the market and facilitating innovation in diverse areas of life, such as health care, food safety, electronics, transport, and aerospace [1]. Nevertheless, with only about 9% of all plastics recycled and 12% incinerated, the vast majority of the plastics ever produced ended up in landfills or the natural environment [2]. Spreading from the deepest seas to the tallest mountains [3, 4], plastic pollution became present in all of the Earth’s habitats. With its negative impacts on ecosystems, human health, and economy, it came to be one of the greatest environmental challenges of our time [5, 6].

The solution to the plastic problem relies on a transition to the circular economy, in which materials stay in use as long as possible and get recovered rather than disposed of once the end of their lifetime is reached [7]. At present, the most viable route for plastic recovery is the physical re-processing of the plastic waste into granulates or new products known as mechanical recycling [8]. As the quality of the resulting recyclates highly depends on the purity of the plastic fractions, a thorough identification and sorting of the incoming waste is required [9]. While manual sorting was the only available option in the past, development of a near-infrared (NIR) technology enabled its automatization resulting in lower recycling costs, higher accuracies, and ultimately, greater amounts of plastics recycled. In the meantime, NIR-based sorting technology became the state of the art in many European countries [10]. Despite its outranging performance, NIR lacks the ability to identify dark and black plastics, rendering these fractions unrecycled. Thus, there is a need for a method which could fill these gaps and enable the current recycling rates to increase.

Over the past few years, laser-induced breakdown spectroscopy (LIBS) has earned an increasing interest in the field due to its ability of delivering a rapid analysis of materials regardless of their thickness or color [11, 12]. In LIBS, a pulsed laser is employed to ablate, atomize, and excite a small portion of the sample, forming a short-lived plasma in the process [13, 14]. As the excited species decay back to their ground levels, the energy of the corresponding transitions is emitted in the form of electromagnetic radiation [15]. Once detected, a complex spectrum carrying information about the elemental composition of the sample is obtained [16]. As different species dominate the plasma at different times, the time of detection greatly affects the information content and appearance of the spectra [17]. Whereas spectra detected in the early plasma stages are dominated by emissions of ions and continuous emission of free electrons (Bremsstrahlung), recombination of these species results in atomic emission lines [18]. At later stages, the energy of the plasma becomes sufficiently low for atoms to recombine, which leads to an emergence of the molecular bands. In addition to the detection time, different experimental parameters governing plasma formation and expansion, such as laser energy or measurement atmosphere, have an effect on the spectral appearance [19].

In the case of organic compounds such as polymers, the amount of useful information provided by the LIBS analysis is restricted by two phenomena: the partial loss of information about the molecular connectivity due to the sample atomization and the high similarity of the LIBS spectra caused by the similar elemental composition of polymers [20]. Nevertheless, subtle variations of the signal intensities related to the different stoichiometric ratios of the polymeric compounds exist and can be detected by means of various chemometric tools, which opens up the possibility of their discrimination [21]. So far, different statistical methods, such as linear and rank correlation [22, 23], mutual distance in the p-dimensional space [24], method of normalized coordinates (MNC) [25], principal component analysis (PCA) [26–28], k-nearest neighbors (k-NN) [29], k-means algorithm [11, 12], partial-least squares discriminant analysis (PLS-DA) [30, 31], supported vector machines (SVM) [32] and artificial neural networks (ANN) [33], were employed for the identification of plastics by means of LIBS. Nevertheless, in all of these studies, only a limited number of different polymer types (mostly around 5 [23, 29, 34], maximally 12 [11, 12]) were employed to establish a classification model. Considering the wide range of plastics available on the market and the high complexity of today’s products, models trained on such a limited number of polymer types might run into problems once applied in the real-life scenario. Moreover, none of the mentioned works addresses the question of model robustness to the presence of polymer additives or pigments such as carbon black, despite the fact that this is presented as one of the main advantages of LIBS over NIR spectroscopy.

Creating a reliable model for the discrimination of many polymer types is, however, not a straightforward task. Considering the signal intensities at the individual wavelengths as coordinates in space, each LIBS spectrum of a polymer can be represented as a p-dimensional point. In order for the discrimination to be efficient, LIBS spectra of one polymer type should show higher similarity to each other than to the spectra of other polymer types. In such case, the points representing a single polymer type would cluster in a defined region of the p-dimensional space, well separated from the remaining clusters, and the inter-cluster variances would dominate over the intra-cluster ones. However, the very small differences in the LIBS spectra of polymers caused by their similar elemental composition are often insufficient to ensure a clear separation of the clusters. With an increasing number of polymer types included in the identification study, the number of clusters contained in the p-dimensional space grows and the probability of cluster overlaps becomes correspondingly high.

This work presents the development of a statistical procedure for a successful discrimination of 20 virgin polymer types using LIBS. By employing novel chemometric approaches, such as spectral descriptors, k-NN cluster purity, and in-house–designed RDF experiments, we are able to overcome the limitations imposed by the spectral similarity and achieve a significant improvement in the resolution of the 20 virgin polymer clusters. Additionally, we demonstrate the robustness of the optimized approach to the presence of carbon black and mixture of common polymer additives, which indicates its potential for the identification of real-life samples.

## Materials and methods

### Chemicals

Polystyrene (PS) and polyacrylonitrile (PAN) in powder form were purchased from Acros Organics (Geel, Belgium). Polyimide P84 (PI) was obtained from HP Polymer GmbH (Lenzing, Austria). The remaining polymer samples were virgin plastic pellets provided by the Faculty of Biology, Chemistry and Earth Sciences, University of Bayreuth, Germany. N-Methyl-2-pyrrolidon (NMP) of p.a. quality, carbon black, and butylated hydroxytoluene were purchased from Merck (Darmstadt, Germany). Irgafos 168, Irganox 1076, and Tinuvin 770 were obtained from BASF (Ludwigshafen, Germany). 2,4-Dibromophenol was obtained from Honeywell Fluka (Schwerte, Germany). High-purity n-doped Si wafer cut into 10 × 10-mm pieces was provided by Infineon Austria AG (Villach, Austria).

### Sample preparation

The set of virgin polymers studied in the present work was comprised of three polymer thin films and eighteen samples of polymer pellets, accounting for 20 different polymer types altogether (Table 1). The thin films were prepared by dissolving PS, PAN, and PI powders in NMP, applying 50 μL of the resulting solution (10–20 wt%) to a 10 × 10-mm high-purity Si wafer (Infineon Austria AG, Villach, Austria) and drying at 100 °C for 4 h to remove the solvent. The polymer pellets (Ø 2–3 mm) were first embedded in an epoxy resin (Epofix Kit, Struers GmbH, Austria) using an embedding medium to polymer ratio of 100:1. The surface of the resulting samples was then polished with a SiC abrasive paper (Struers GmbH, Austria) until a smooth horizontal cross section suitable for the LIBS analysis was obtained.

**Table. 1:**
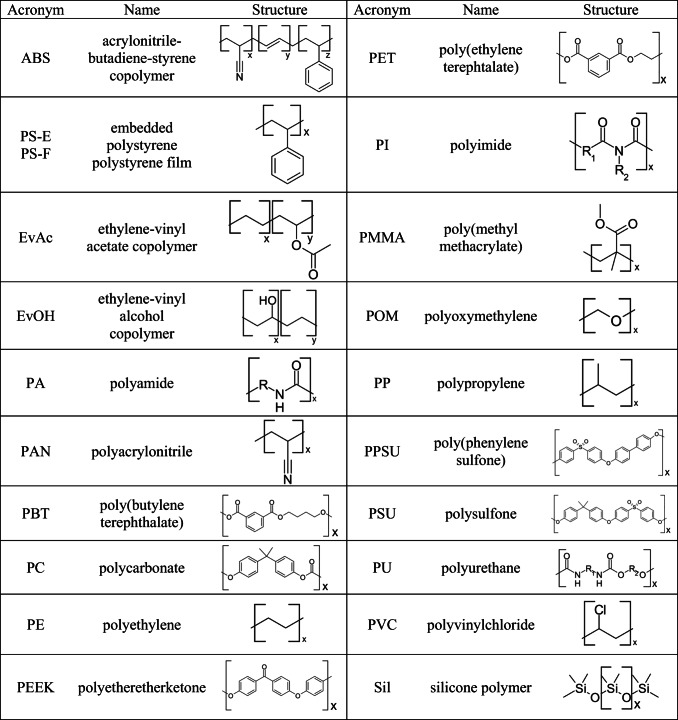
Polymer types used in this work. PAN and PI were analyzed as thin films, the remaining polymers as embedded pellets. PS was used both embedded and as a film (PS-E and PS-F)

In addition to the virgin polymers, two types of polymer-additive samples were prepared by combining the solutions of PS, PAN, and PI with carbon black or with a combination of polymer additives (Table 2). The prepared suspensions were thoroughly homogenized in an ultrasonic bath for 1 h, vortexed for 45 s, and applied to the surface of a Si wafer. The subsequent steps were identical with the preparation of virgin polymer thin films.

**Table. 2:** List of compounds used for the preparation of the polymer-additive samples. The content of the individual additives was selected according to the reference values found in the literature [35]

Sample	Name	Type/function	Concentration [wt%]
Carbon black	Carbon black	Pigment	0.5
Mix of additives	Butylated hydroxytoluene	Antioxidant	0.1
	2,4-Dibromophenol	Flame retardant	0.1
	Irgafos 168	Antioxidant	0.1
	Irganox 1076	Processing stabilizer/antioxidant	0.1
	Tinuvin 770	UV absorber	0.1

### LIBS analysis

The LIBS analysis was carried out using a commercially available LIBS J200 system (Applied Spectra, Sacramento, CA) supplied with Axiom 2.0 software. A Q-switched Nd:YAG laser operating at the fourth harmonics of 266 nm, pulse duration of 5 ns, and 10 Hz repetition rate was used for the sample ablation and excitation. A system of collection optics connected to optical fibers was used to collect and transmit the emitted light to a 6-channel Czerny-Turner spectrometer. The total wavelength region covered by this experimental set-up ranged from 188 to 1048 nm.

Using a motorized x-y-z stage moving at a constant velocity of 1 mm/s, laser beam diameter of 100 μm, and horizontal line scan pattern covering an area of 1.2 mm × 1 mm, 120 single-shot spectra were recorded for each of the virgin polymer samples. In order to avoid interference, the individual measurements were carried out at a distance of 100 μm to the preceding measurement. Every sample was analyzed under 24 different experimental conditions involving systematic changes of laser energy (1.8, 2.4, and 3 mJ) and gate delay (0.1, 0.4, 0.7, and 1 μs) under two different atmospheres (see Table 3 for further details). In the case of the embedded samples, a pre-ablation step employing a laser energy of 1.8 mJ was used to remove possible surface contamination originating from the sample preparation.

**Table. 3:** Experimental parameters used for the investigation of the 20 virgin polymer types. Alteration of one parameter at a time resulted in 24 unique combinations of gate delay, laser energy, and atmosphere

Gate delay	0.1 μs	0.4 μs	0.7 μs	1 μs
Laser energy	1.8 mJ	2.4 mJ	3 mJ	
Atmosphere	Argon	Air		

The polymer additive samples were analyzed using an optimized set of experimental conditions described in “Optimization of the LIBS parameters” in the section “Results and discussion.” In this case, 104 single-shot spectra covering a total area of 2.6 × 0.4 mm were acquired.

### Data analysis

The acquired data was imported to the multisensor imaging tool Epina ImageLab, Release 3.30 (Epina GmbH, Retz, Austria), which enables a fast extraction of chemically relevant information from raw spectra by means of “spectral descriptors.” These represent single intensities, sums of intensities, or more complex mathematical functions calculated from specific regions of the spectra defined by the user. In the present work, 4 types of descriptors designated as ABL, PRW, PBL, and PLV (cf. Table 4) were used. The advantages of using spectral descriptors in the field of FTIR/Raman have been demonstrated in the previous work [36] and are related to an effective reduction of the variable space while preserving the most relevant chemical information for the given analysis.

**Table. 4:**
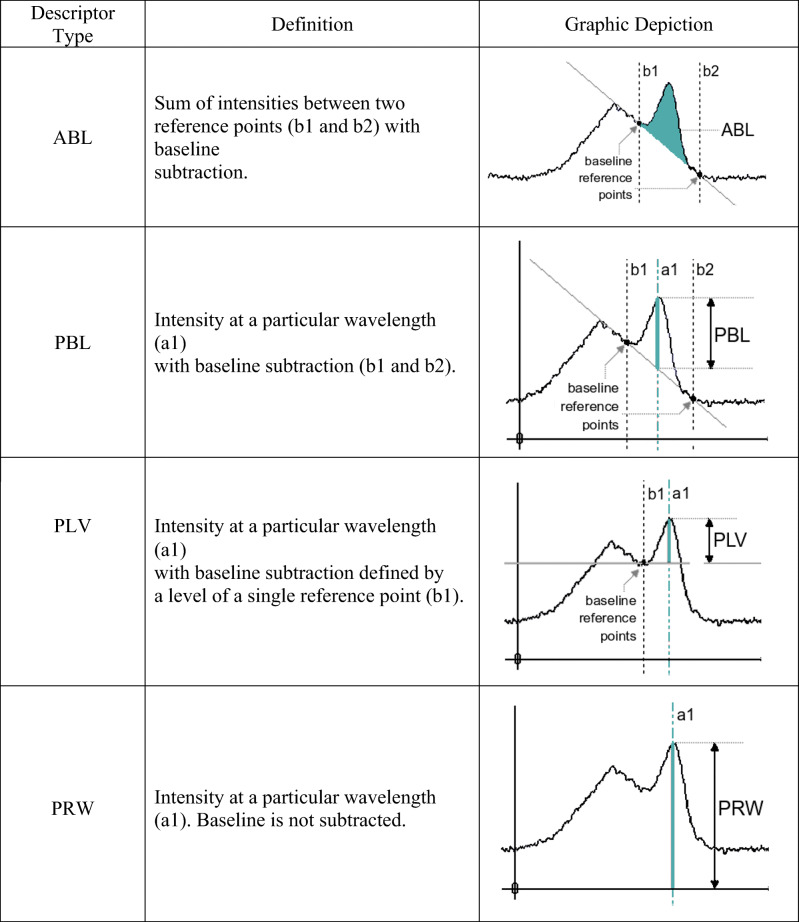
Types of spectral descriptors used in the present work [36]

Inspired by the previous work on polymer classification [26], first, a basic set of spectral descriptors representing the most characteristic features of polymers was established. As shown in Table 5, it comprised of 10 ABL descriptors related to the emission signals of C, H, and O, as well as to the molecular emissions of C2 and CN. Additionally, signals of Cl and Si were included due to the presence of PVC and silicone in the studied polymer set.

**Table. 5:** Basic set of spectral descriptors

Emission signal	Integrated wavelength range [nm]
C (I)	192.59–193.41
C (I)	247.44–248.44
Si (I)	250.17–253.77
Si (I)	287.49–288.89
CN violet band	387.42–388.65
C2 Swan band delta ν-1	473.22–474.24
C2 Swan band delta ν	516.23–516.75
H (I)	650.19–663.74
O (I)	776.51–778.42
Cl (I)	837.16–838.35

Pre-processing of the virgin polymer data was comprised of a random selection of 20 single-shot spectra per measurement (out of the 120 spectra acquired), normalization of each spectrum to a constant sum of 1000, extraction of the spectral descriptor information and standardization of the resulting data (mean = 0, std. dev. = 1). Thereby, the dimensionality of the variable space was reduced from the original 12,288 dimensions to only 10, which has an overall positive effect on the subsequent analysis, as it addresses the problems of overfitting and curse of dimensionality and substantially reduces the computation times. The multivariate analysis of the pre-processed data—the principal component analysis (PCA), k-nearest neighbors (k-NN), and hierarchical cluster analysis (HCA)—was carried out using Epina DataLab Rel.4.0 (Epina GmbH, Retz, Austria). k-NN was performed with 5 nearest neighbors and majority voting, HCA with Ward’s method of linkage. Both of the algorithms employed Euclidean measure of distance in the p-dimensional space.

In the case of the polymer-additive experiments, all of the 104 single-shot spectra per sample were subjected to data processing comprised of spectrum normalization to constant sum and extraction of the optimized descriptor information (detailed description provided in “Optimization of the spectral descriptors/variablesS12” of the “Results and discussion” section). The resulting data set was standardized (mean = 0, std. dev. = 1) and subjected to the PCA analysis.

## Results and discussion

As outlined in the introduction, the greatest challenge to the identification of many polymer types by means of LIBS is the high similarity of their spectra resulting in extensive cluster overlaps. Therefore, the main goal of this work was to improve the resolution of the 20 virgin polymer clusters in the p-dimensional space. This could be achieved by a systematic optimization of two sets of parameters having an influence on the mutual separation of the clusters: the set of experimental conditions affecting the appearance and thus the location of the polymer spectra in the space and the set of spectral variables used as a basis of the space. In the first part of the study, a concept of the k-NN-based cluster purity was introduced to investigate the effect of three experimental parameters (laser energy, gate delay, and atmosphere) on the identification potential of the 20 polymer types. The dataset delivering the best results was then subjected to a PCA and k-NN analysis, which provided a deeper insight into the relationships of the clusters in the p-dimensional space. The second part of the study aimed for further improvement of the cluster resolution by identification of a spectral descriptor set providing the best discrimination of the studied polymers. This could be achieved by employing an in-house-designed RDF experiment and a forward selection algorithm governed by the cluster purity. The final resolution of the polymer clusters was then studied by means of PCA, k-NN, and HCA. Eventually, PCA analysis of polymer samples containing different additives was performed to investigate the robustness of the developed discrimination method.

### Optimization of the LIBS parameters

**Fig. 1:**
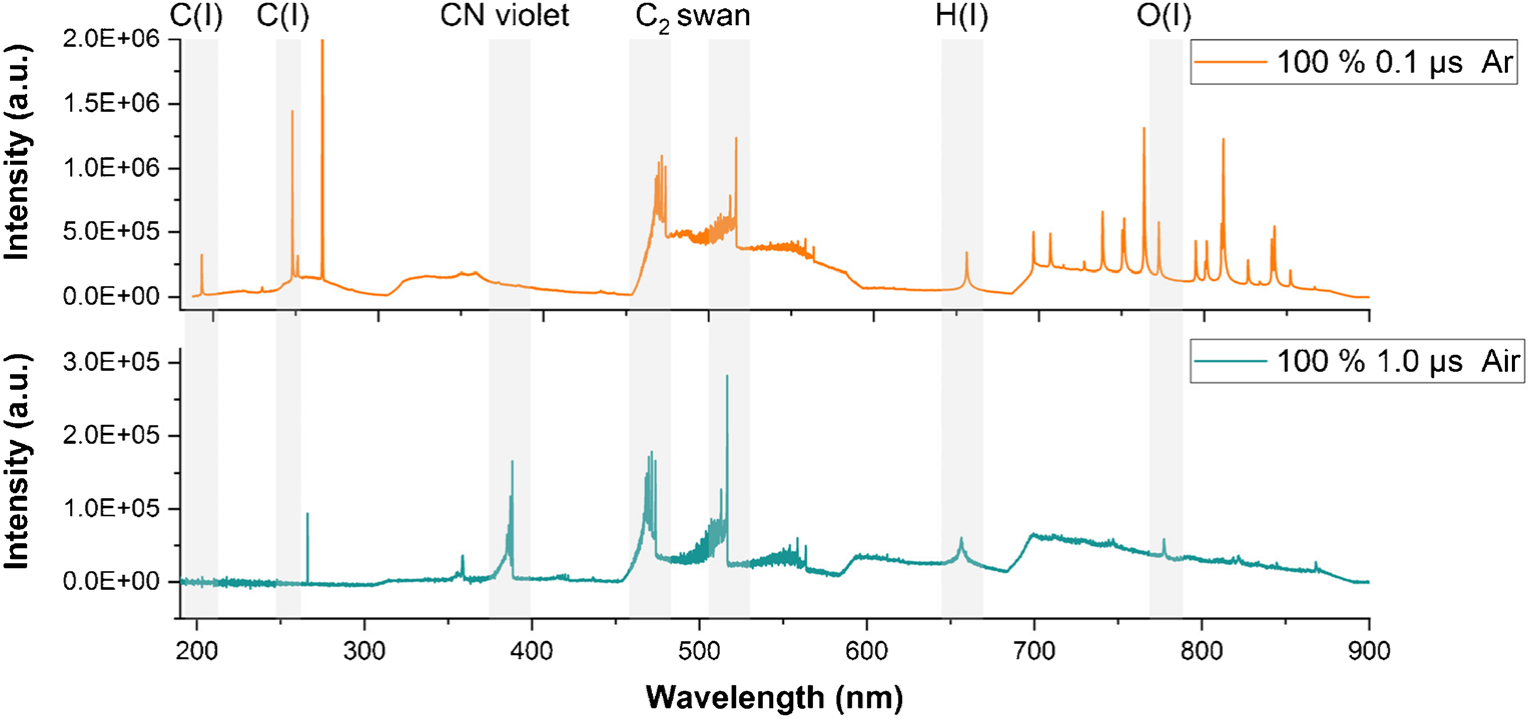
LIBS spectra of PS obtained under two different experimental settings. Gray regions highlight the polymer-specific signals

As demonstrated by Fig. 1, the choice of experimental conditions has a profound effect on the information delivered by the LIBS analysis reflected by the spectral appearance. Whereas the spectrum acquired at a shorter gate delay and argon atmosphere provides information about the atomic emissions of carbon directly related to the polymer, this type of information is missing in the spectrum acquired with longer gate delay and air. In this case, the carbon content is partially encoded in the CN band arising from the recombination of carbon species stemming from the sample with molecules present in the surrounding air [37]. Another difference worth highlighting is the higher total intensity of the spectrum acquired at shorter gate delay.

**Fig. 2:**
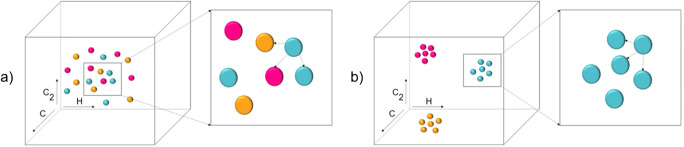
Use of the k-NN algorithm for assessment of the cluster purity; **a** low cluster purity—the k-nearest neighbors of each data point/sample belong to random classes, **b** high cluster purity—the k-nearest neighbors of each data point belong to the same class as the data point

The presented figure demonstrates two sets of conditions studied for a single polymer type. Nevertheless, the current work investigated 20 polymer types under 24 different combinations of laser energy, gate delay, and atmosphere. Thus, a more elaborate tool was required to infer the effect of the experimental conditions on the spectral appearance influencing the positioning and clustering of the polymer points in the data space. Therefore, a concept of total cluster purity based on the k-NN algorithm was introduced. An intuitive representation of this idea is depicted in Fig. 2: if the k-nearest neighbors of each polymer within each class have the same identity as the polymer itself, the clusters are expected to be pure and the identification of the polymers efficient, whereas if the identity of the k-nearest neighbors is random, the total cluster purity is expected to be low and the polymer identification poor.

In practice, cluster purity can be obtained using the following procedure:

1. Determine the k-nearest neighbors of each data point.
2. Estimate the class labels of the neighbors using the k-NN algorithm.
3. Track the frequencies of occurrence of the individual sample (*Y*_*j*_ )-neighbor (${\hat{Y}}_i$) combinations in the corresponding cells of the k-NN contingency table (*n*_*ij*_).

**Table:** 

${\hat{Y}}_i\backslash {Y}_j$	*Y*_1_	*Y*_2_	…	*Y*_*s*_	Sums
${\hat{Y}}_1$	*n*_11_	*n*_12_	…	*n*_1*s*_	*a*_1_
${\hat{Y}}_2$	*n*_21_	*n*_22_	…	*n*_2*s*_	*a*_2_
⋮	⋮	⋮	⋱	⋮	⋮
${\hat{Y}}_s$	*n*_*s*1_	*n*_*s*2_	…	*n*_*ss*_	*a*_*s*_
Sums	*b*_1_	*b*_2_	…	*b*_*s*_	$\sum \limits_{i,j}{n}_{i,j}=N$

4. Use results of the contingency table to calculate the adjusted Rand index value, RI_adj_ [38], according to the following equation:

$$ \mathrm{R}{\mathrm{I}}_{\mathrm{adj}}=\frac{\sum_{i,j}\left(\genfrac{}{}{0pt}{}{n_{ij}}{2}\right)-\left[{\sum}_i\left(\genfrac{}{}{0pt}{}{a_i}{2}\right){\sum}_j\left(\genfrac{}{}{0pt}{}{b_j}{2}\right)\right]/\left(\genfrac{}{}{0pt}{}{n}{2}\right)}{\frac{1}{2}\left[{\sum}_i\left(\genfrac{}{}{0pt}{}{a_i}{2}\right)+{\sum}_j\left(\genfrac{}{}{0pt}{}{b_j}{2}\right)\right]-\left[{\sum}_i\left(\genfrac{}{}{0pt}{}{a_i}{2}\right){\sum}_j\left(\genfrac{}{}{0pt}{}{b_j}{2}\right)\right]/\left(\genfrac{}{}{0pt}{}{n}{2}\right)} $$

The resulting adjusted Rand index value represents the extent of cluster purity (low values correspond to randomly mixed clusters, a value of 1 to perfectly pure ones). In order to account for the possible signal fluctuations among the single-shot spectra, the process of random data selection, descriptor extraction, and calculation of the cluster purity was performed 100 times. The resulting mean cluster purities obtained under the individual experimental conditions are summarized in Table 6.

**Table. 6:** RI_adj_ values obtained with different combinations of laser energy and gate delay for argon (left) and air (right). Bold: best set of experimental conditions

Laser Energy [%]	Argon				Air			
	Gate delay [μs]				Gate delay [μs]			
	0.1	0.4	0.7	1	0.1	0.4	0.7	1
60	0.6525	0.5282	0.3158	0.2409	0.4650	0.3090	0.2485	0.1433
80	0.7314	0.6936	0.4866	0.3250	0.5646	0.3693	0.2460	0.2028
100	**0.7558**	0.7501	0.5594	0.4354	0.5382	0.4266	0.3086	0.2343

As the trends in the cluster purity show, all of the investigated parameters (laser energy, gate delay, and atmosphere) had an effect on the cluster separation and thus on the identification potential of the 20 virgin polymer types. In the vast majority of experiments, an increase in the laser energy resulted in an improved cluster purity, which correlates well with the fact that the laser energy of 3 mJ delivered the highest signal-to-noise ratio. The most significant changes of the cluster purity were related to the alterations of gate delay, which clearly demonstrates the importance of its optimization. In the case of polymers, this process typically involves finding a compromise between shorter gate delays delivering atomic signals of high intensities and longer gate delays providing information about the arising molecular emissions. In the present work, the greatest identification potential was achieved at the earliest gate delay (0.1 μs), which can be explained by a high intensity of the carbon atomic emission line and a simultaneous presence of the C2 molecular bands in the corresponding spectra. The presence of the C2 signals at comparably short gate delays was reported in previous work dealing with LIBS analysis of organic compounds [39] and attributed to the fractionation of larger carbon clusters directly ejected from the sample rather than the recombination of C atoms upon plasma expansion and cooling.

Comparing the two atmospheres, argon delivered better results than air in all of the investigated cases. This might be explained by the presence of collision partners contributing to the formation of mixed polymer-air species, such as CN [37], which might lead to a depletion of the polymer-specific species crucial for a successful identification.

**Fig. 3:**
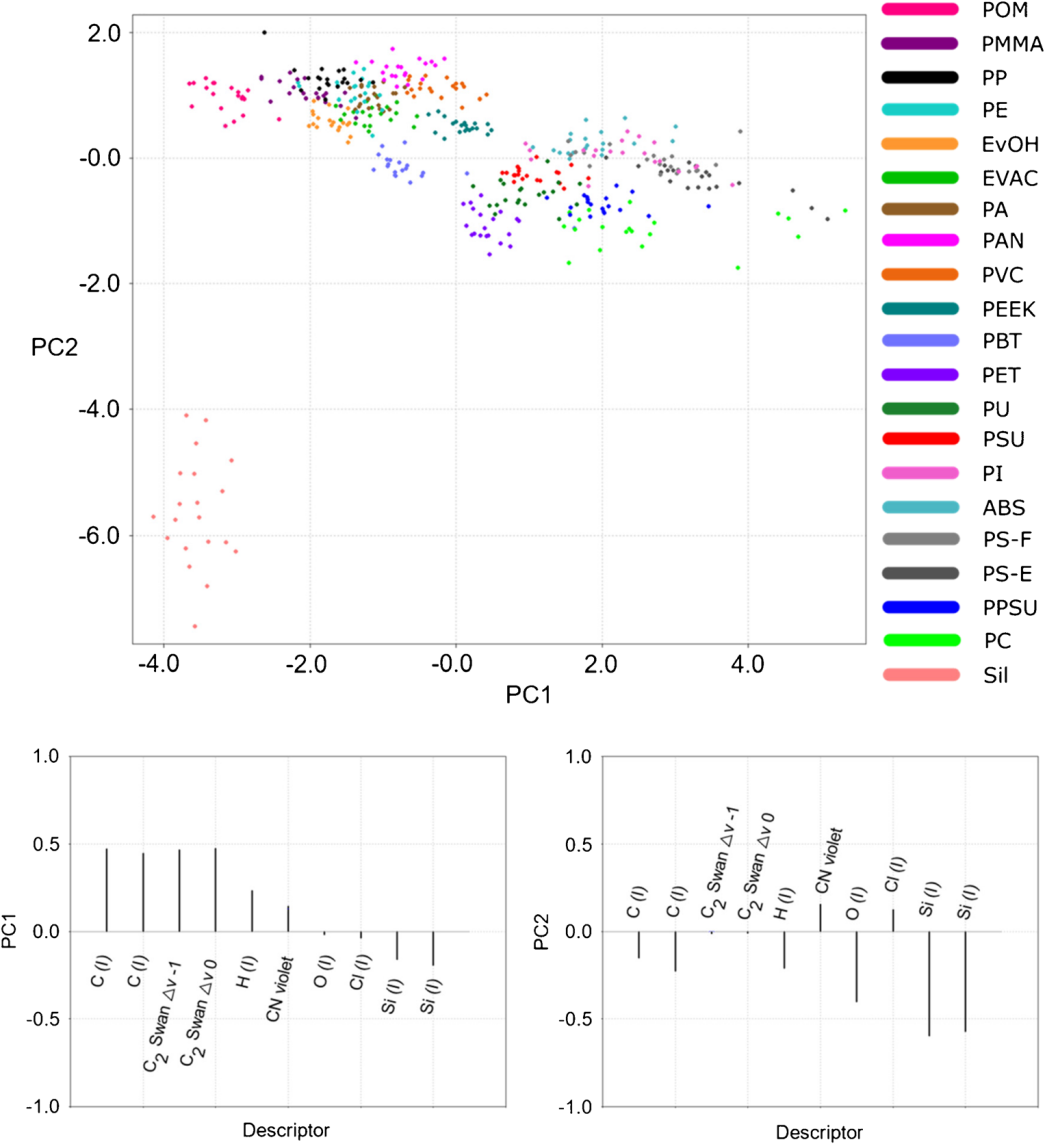
Basic set of spectral descriptors: separation of the polymer clusters in the plane defined by principal component 1 (horizontal axis) and principal component 2 (vertical axis); LIBS conditions: 100% laser energy, 0.1 μm gate delay, argon atmosphere

To sum up, the best resolution of the 20 virgin polymer clusters studied in the present work could be achieved using the highest laser energy (3 mJ), shortest gate delay (0.1 μs), and argon atmosphere (Table 6, bold value). In order to obtain a visual impression of the data, a PC1/PC2 score/score plot accounting for 62.61% of the total data variance is presented in Fig. 3 together with the corresponding loadings.

**Fig. 4:**
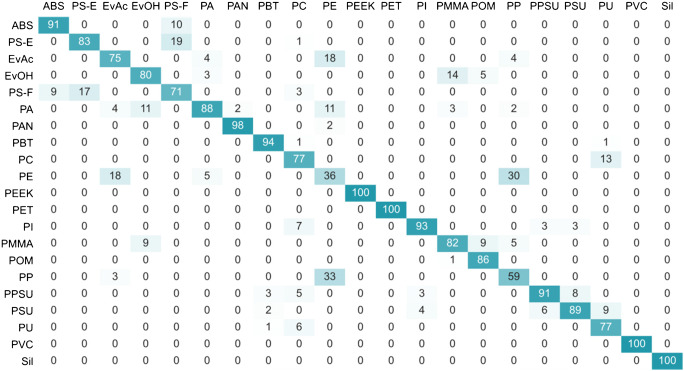
Basic set of spectral descriptors: k-NN contingency table providing the information about the identity of the 5 nearest neighbors to all datapoints from one polymer type; LIBS conditions: 100% laser energy, 0.1 μm gate delay, argon atmosphere

As the PC1 loadings imply, the separation of the polymers along the PC1 axis was mostly governed by the intensities of the atomic and molecular emissions of carbon related to the native molecular bonds (C–C and C=C) of the polymers. Whereas polymers such as POM with no native C–C bonds or PE with saturated C–C backbone and simple H substituents reached lower scores and ended up in the left region of the plot, the scores of the polymers containing aromatic rings, such as PC or PS, were comparably higher, which resulted in their rightward position. These findings correlate well with the findings of Grégoire et al. [28] published previously. Introduction of the second PC axis resulted in a complete separation of the silicone group from the remaining classes and an improved resolution of polymers with similar molecular structures. Despite this fact, the overlap of many polymer classes remained high, which implied a rather poor potential of their identification. Interestingly, the two types of polystyrene samples involved in the study (polystyrene film—PS-F and embedded polystyrene pellets—PS-E) seem to occupy slightly different regions of the space, which might allow for their discrimination. A reason for this could be the different ablation behavior of the two samples. As the PC1/PC2 plot provides only a 2-dimensional representation of the 10-dimensional data, the PCA results were complemented with a k-NN contingency analysis providing detailed information on the surroundings of the individual polymers in the basic descriptor space (Fig. 4).

According to this, only the clusters of PEEK, PET, PVC, and Sil were completely pure. Other classes of polymers sharing common structural features, such as PMMA and EvOH, PU and PC, or ABS and PS-F, were prone to cluster overlaps leading to decreased identification rates. The discrimination among PE, PP, and EvAc (containing 14 wt% Ac) was shown to be the most problematic, which can be attributed to the high degree of their spectral similarity. As the total cluster purity achieved in this data set, representing the optimal experimental conditions, was rather poor (RI_adj_ = 0.7558), an additional means of improving the resolution of the 20 virgin polymer clusters was required.

### Optimization of the spectral descriptors/variables

The approach presented in this section relies on redefinition of the variable space in such a way that an optimal cluster separation becomes guaranteed. In order to find a combination of variables fulfilling this condition, the basic set of variables was augmented by additional descriptors in a two-step process:

At first, 30 descriptors of three additional types were generated using the same spectral regions as in the basic descriptor set (Table 5). They represented raw signal intensities with (PBL and PLV) and without (PRW) a baseline correction. In the case of the PBL descriptors, the baseline was defined by an average of 5 neighboring (detector) pixels to the reference points, whereas in the case of the PLV descriptors, a fixed baseline at 187.98 nm was selected due to the lack of interfering emissions across the investigated range of samples. At the end of this step, the augmented descriptor set was comprised of 40 descriptors (ABL, PRW, PLV, PBL) representing the most characteristic emission features of the studied polymers.

The second step involved the identification of additional spectral regions important for the discrimination of the 20 polymers. This could be achieved by using an in-house-designed random forest (RDF) experiment, in which the studied polymers were divided into two classes depending on the presence or absence of certain chemical substructures. A random forest classifier was trained to discriminate these two classes, and the resulting variable importance was used to identify spectral regions supporting their discrimination.

In this work, two such experiments were performed, one aiming to identify the spectral regions important for the discrimination of the aromatic and non-aromatic polymers and one for the discrimination of the N-containing and N-lacking polymers. Each of these RDF experiments (no. of trees = 75, *R* = 0.5) was carried out twice, once using a raster of raw signal intensities (PRW descriptors) with a regular spacing of 0.3 nm and once using a raster of peak areas (ABL descriptors) with a regular spacing of 0.9 nm. By appending all descriptors with a high variable importance to the augmented descriptor set, a total of 84 spectral descriptors relevant for the discrimination of the 20 virgin polymers were obtained.

**Fig. 5:**
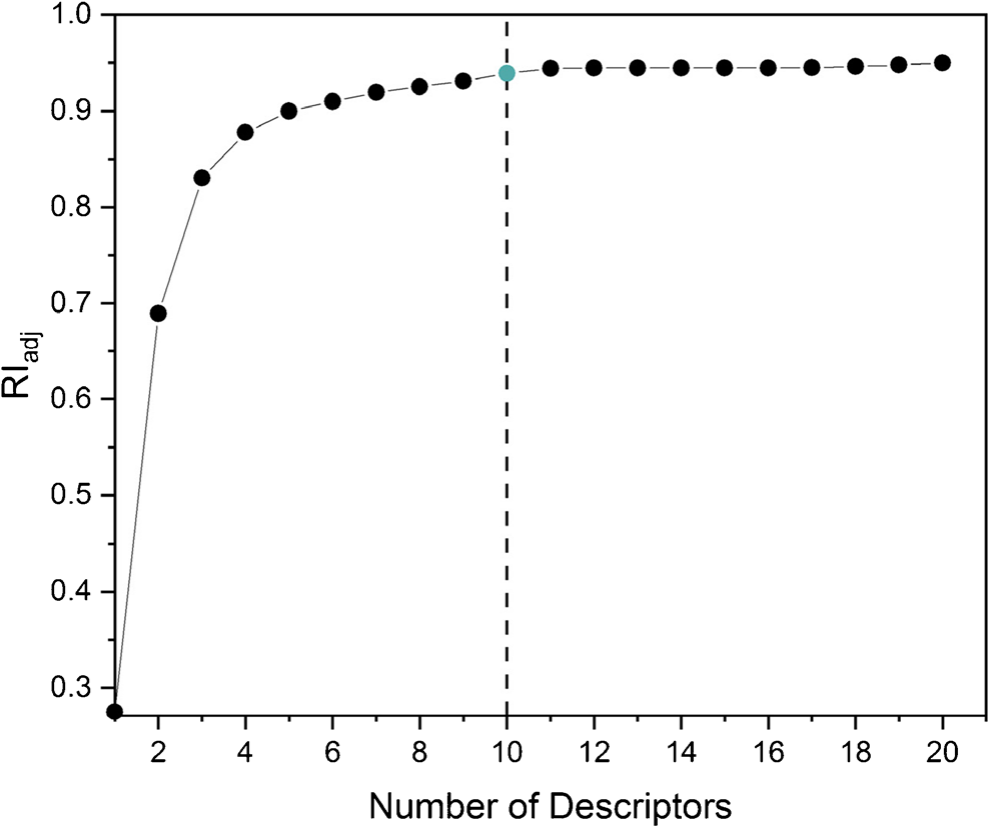
Forward selection algorithm governed by the cluster purity (RI_adj_) used for pruning of the augmented descriptor set. Dotted line, number of spectral descriptors used to establish the optimized descriptor set

Finally, this set of 84 descriptors was pruned by applying a simple forward selection algorithm (Fig. 5), keeping only those descriptors which contribute most to the improvement of the cluster purity. In order to ensure the stability of the selected descriptor set, the pruning was repeated with 10 different datasets obtained by random sampling of the measured data.

As no significant improvements of the cluster purity could be observed with more than 10 descriptors (Fig. 5, blue point), the forward selection was stopped after the 10th descriptor resulting in a set of 10 optimized descriptors. As presented in Table 7, the optimized set contained 4 of the original ABL descriptors representing the baseline-corrected peak areas of O, C, and H and of 6 descriptors of PBL, PLV, and PRW types representing the baseline-corrected as well as baseline-non-corrected peak intensities from the emission regions of C, O, Si, C2, and CN.

**Table. 7:** Optimized set of spectral descriptors; bl: baseline

Importance	Emission signal	Descriptor type	Integrated wavelength range [nm]
1	O (I)	ABL	776.51–778.42
2	C (I)	ABL	247.44–248.44
3	C (I)	ABL	192.59–193.41
4	C2 Swan band delta ν-1	PRW	469.38
5	C (I)	PLV	247.87 (bl, 187.98)
6	H (I)	ABL	650.19–663.74
7	O (I)	PBL	776.01–779.14
8	Si (I)	PRW	251.60
9	CN violet band	PBL	383.39–389.72
10	C2 Swan band delta ν	PLV	516.61 (bl, 187.98)

**Fig. 6:**
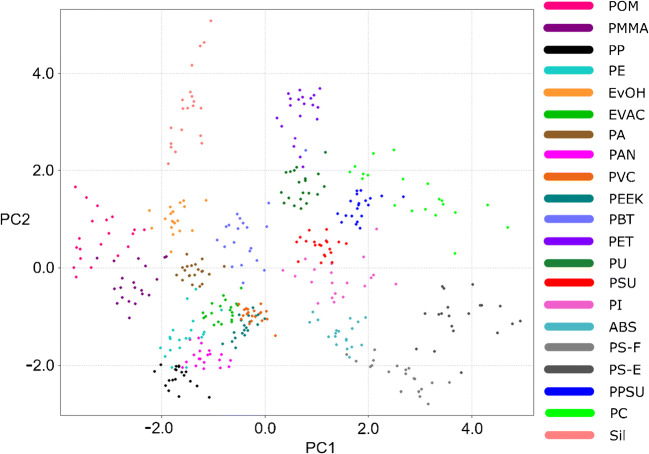
Optimized set of spectral descriptors: separation of the polymer clusters in the plane defined by principal component 1 (horizontal axis) and principal component 2 (vertical axis). LIBS conditions: 100% laser energy, 0.1 μm gate delay, argon atmosphere

The ability of the optimized descriptor set to provide an improved cluster resolution was first examined by means of PCA. In contrast to the basic set of descriptors, almost complete separation of the polymer classes across the PC1/PC2 plane could be achieved (Fig. 6).

**Fig. 7:**
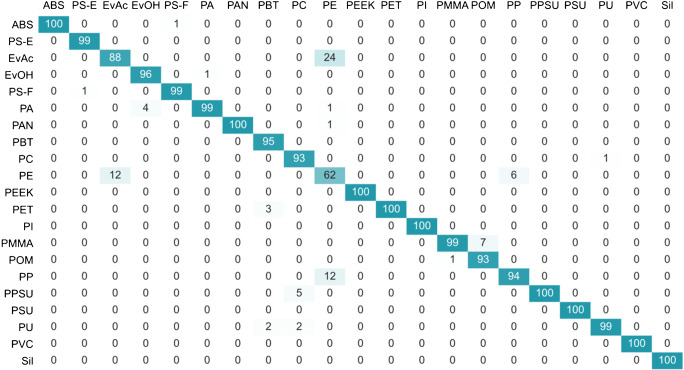
Optimized set of descriptors: k-NN contingency table providing the information about the identity of the 5 nearest neighbors to all datapoints from one polymer type; LIBS conditions: 100% laser energy, 0.1 μm gate delay, argon atmosphere

As before, the PCA results were complemented by a contingency table of k-NN providing a detailed information on the surroundings of the polymers in the optimized descriptor space (Fig. 7).

Whereas in the case of the basic descriptor set, only 4 polymer classes were completely pure, after the redefinition of the space, this number increased to 9. Moreover, the problems of the discrimination between certain polymer classes, such as ABS and PS-F, PU and PC, or PSU and PPSU, were either greatly reduced or completely eliminated. Despite significant improvements in the identification of PP, the discrimination between PE and EvAc (Ac 14% w/w) still remained problematic, which can be attributed to a very high degree of their structural similarity. All in all, the optimization of the spectral descriptors resulted in an increase of the cluster purity from 0.756 to 0.925.

**Fig. 8:**
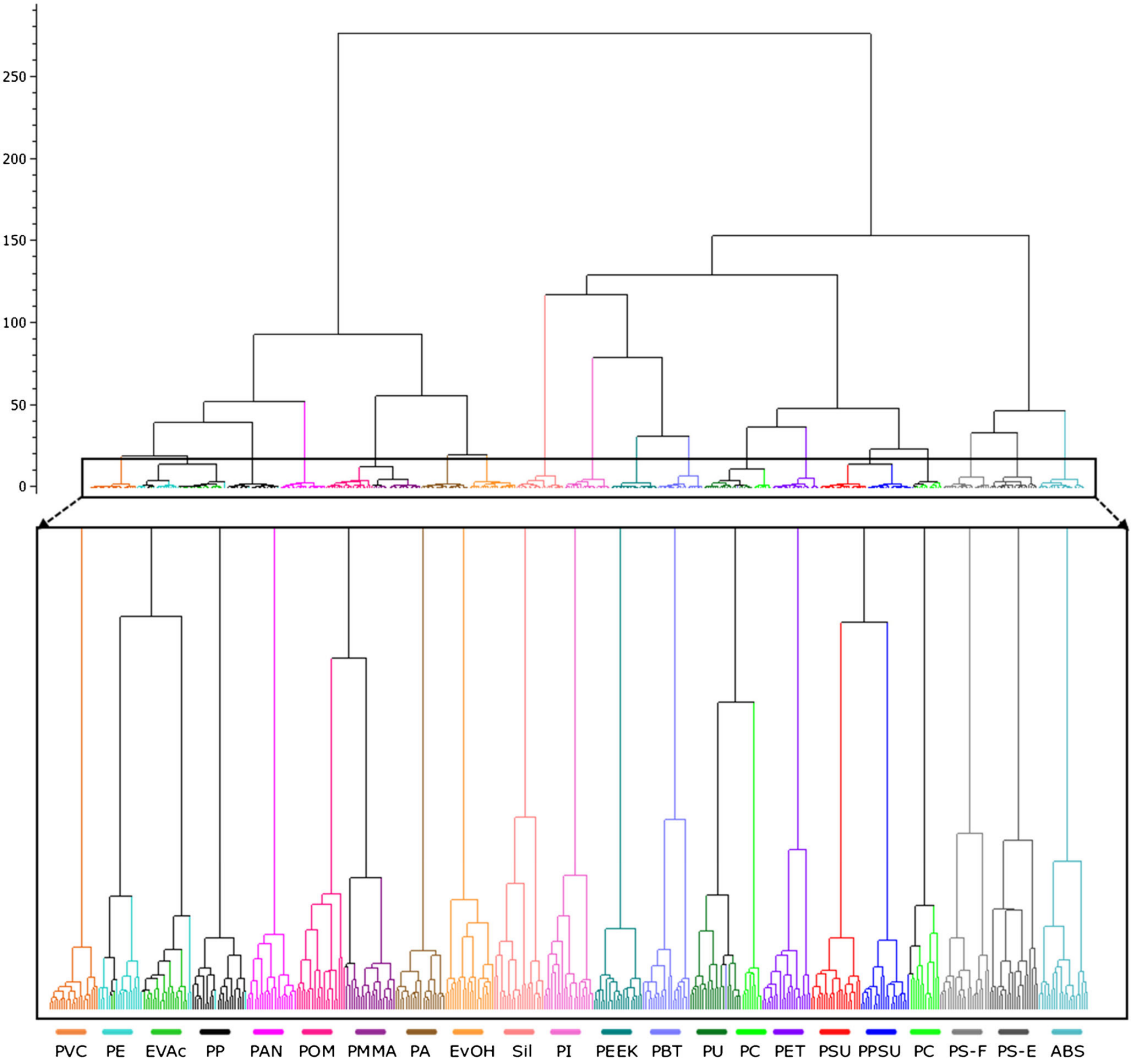
Optimized set of descriptors: dendrogram resulting from the HCA analysis; LIBS conditions: 100% laser energy, 0.1 μm gate delay, argon atmosphere

Eventually, the performance of the optimized set was examined by means of the hierarchical cluster analysis (HCA) (Fig. 8). In total, 22 polymer clusters were identified instead of the 21 present in the data. The false division of the PC cluster into two separate classes might be caused by its spread-out nature and a close proximity of the different cluster regions to PU and PPSU classes in the optimized descriptor space. Overall, the mutual relationships of the HCA clusters were in a good agreement with the results obtained from the PCA and k-NN. The high degree of the cluster purity further proved the ability of the optimized set to provide a better cluster resolution, allowing for an efficient identification of the 20 virgin polymer types.

### Influence of polymer additives

**Fig. 9:**
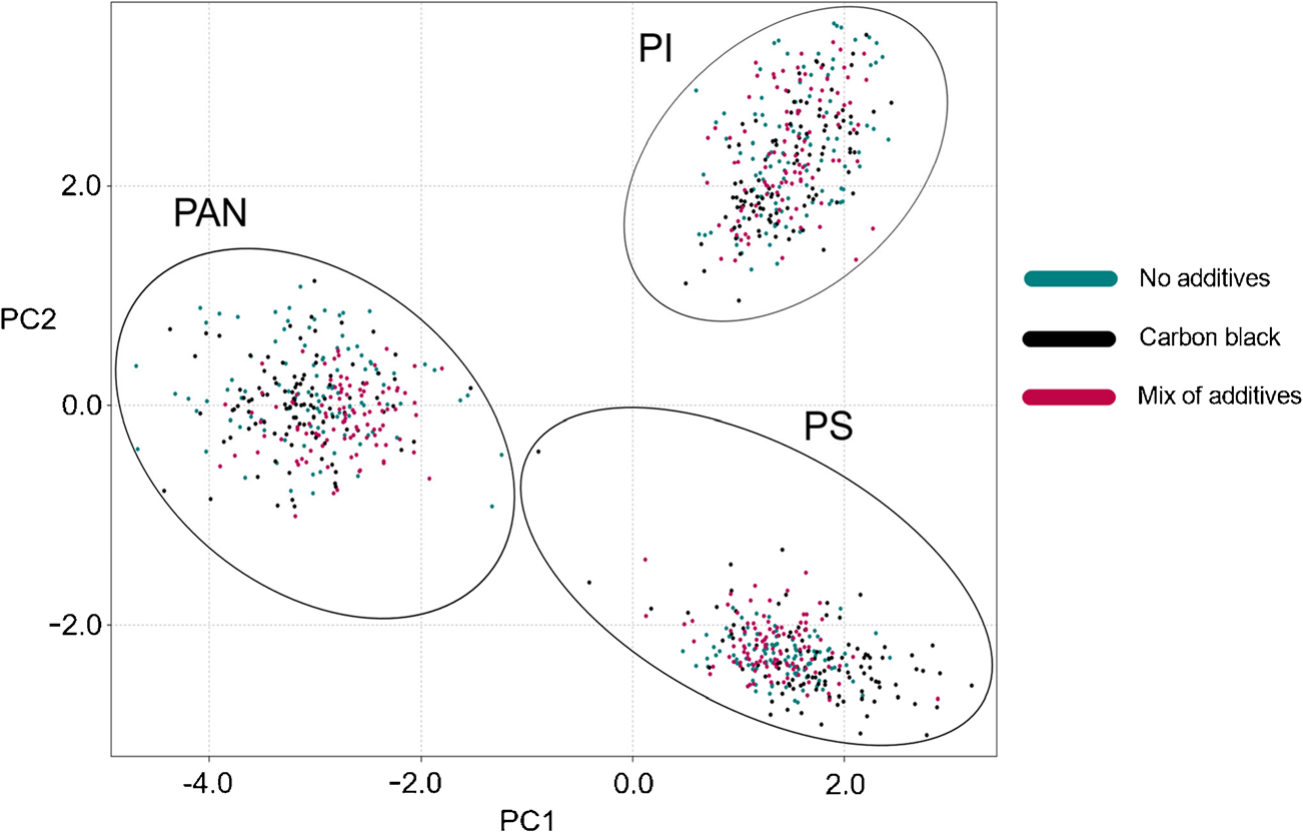
The presence of additives does not interfere with the discrimination of the polymers. The presented data was acquired and processed using the optimized set of experimental conditions and spectral descriptors

In order to investigate the robustness of the developed discrimination approach to the presence of polymer additives, samples of PS, PAN, and PI with different additive composition (no additives, carbon black, and mix of additives (Table 3)) were subjected to LIBS analysis under the optimized set of experimental conditions. By extracting the optimized set of descriptors from the acquired spectra and subjecting the resulting dataset to the PCA analysis, 3 clear clusters corresponding to the 3 polymer types were obtained (Fig. 9). As the PC1/PC2 score/score plot accounting for 82.56% of the data variance shows, the data points belonging to a single polymer type were homogeneously distributed within the polymer cluster regardless of the additive presence, which clearly demonstrates the robust nature of the optimized discrimination approach.

## Conclusion

The present work demonstrates the possibility of using LIBS for the identification of 20 virgin polymer types, which is, to the best of our knowledge, the highest number reported in the literature so far. The problem of the extensive cluster overlaps related to the high spectral similarity of polymers could be resolved by a two-step optimization of the cluster purity based on the k-NN algorithm. An initial improvement of the cluster resolution could be achieved by identifying the set of experimental conditions (laser energy, gate delay, and atmosphere) delivering the highest cluster purity. As the dataset achieving the best results (3 mJ laser energy, 0.1 μs gate delay, and argon atmosphere) still suffered major cluster overlaps (PCA plot, RI_adj_ = 0.756), it was subjected to a second optimization step aiming at an identification of spectral descriptors with the highest significance for the discrimination of the 20 virgin polymer types. In the process, two RDF experiments were employed to find new spectral regions of interest and a k-NN-governed forward selection algorithm was used for the final selection of variables. The optimization of the variable space resulted in a significant improvement of the cluster resolution, which was proved by the PCA, k-NN, and HCA analyses of the corresponding data. All in all, the cluster purity could be improved to a RI_adj_ value of 0.925, which demonstrates the possibility of using LIBS and chemometrics for the identification of 20 virgin polymer types. Moreover, using the optimized experimental design, it was possible to discriminate not only virgin polymer samples, but also polymers containing additives, which indicates the potential of the developed approach for the identification of real-life samples.
